# Mesenchymal Stem Cell-Derived Extracellular Vesicles: Immunomodulatory Effects and Potential Applications in Intervertebral Disc Degeneration

**DOI:** 10.1155/2022/7538025

**Published:** 2022-02-18

**Authors:** Shaojun Hu, Hongyuan Xing, Jiangnan Zhang, Zemin Zhu, Ying Yin, Ning Zhang, Yiying Qi

**Affiliations:** ^1^Department of Orthopedics, 2nd Affiliated Hospital, School of Medicine, Zhejiang University, 88 Jiefang Road, Zhejiang, 310009 Hangzhou, China; ^2^Department of Orthopedics Surgery, Wenling 1st People's Hospital, 190 Taiping South Road, Wenling, 317500 Zhejiang, China; ^3^People's Hospital of Changxing County, Changxing, 313100 Zhejiang Province, China; ^4^Department of Gastroenterology, Affiliated Zhongda Hospital of Southeast University, Nanjing, 210000 Jiangsu, China

## Abstract

Intervertebral disc (IVD) degenerative disease is a common health problem worldwide. Administration of mesenchymal stem cells (MSCs) in intervertebral disc degeneration (IVDD) has been widely explored in recent years. However, transplantation of MSCs is restricted by several factors. Currently, paracrine signaling is one of the main mechanisms by which MSCs play a therapeutic role in disc regeneration. Extracellular vehicles (EVs) are the main paracrine products of MSCs. They show great potential as an effective alternative to MSCs and play immunomodulation roles such as anti-inflammatory effects, antioxidative stress, antiapoptosis, and antiextracellular matrix (ECM) degradation during treatment of IVDD. This review focuses on the immunomodulatory effect of MSC EVs and their potential applications.

## 1. Introduction

Low back pain is a disease that causes high rates if disabilities globally [1]. Intervertebral disc degeneration (IVDD) is a major cause of low back pain. However, the cause of IVDD has not been fully elucidated. Mechanical overloading [2], defective nutrient supply, cell death [3], and progressive fibrosis [4] are associated with progression of IVDD. Studies report that degradation of extracellular matrix is the main factor that causes IVDD and is characterized by increased inflammatory mediators expression [5, 6]. Therefore, several therapeutic approaches for treatment of IVDD are based on reducing matrix degradation and downregulating expression of inflammatory factors within the IVD. Cell therapy has widely been explored as an alternative for treatment of IVDD [7–10]. Therefore, several novel therapies including mesenchymal stem cell (MSC) transplantation have been developed [11]. MSCs are a group of cells with multidirectional differentiation and self-expansion abilities. Studies report that MSCs can differentiate into NP-like cells [12–14]. Therefore, the role of MSCs in disc tissue engineering has been widely explored. A major limitation in IVDD treatment is that its inherently avascular and high osmotic pressure harsh environment limits efficacy of therapeutic agents. Moreover, the dense fibrous ring envelope makes limits entry of cells in large quantities [2, 15]. Notably, only a small percentage of MSCs is eventually integrated into the damaged area during the repair process thus promoting tissue regeneration. Therefore, high efficacy of the treatment is not achieved. Recent studies report that paracrine secretion of mesenchymal stem cells is an important mode of tissue repair [16–18]. Extracellular vehicles (EVs) are the main components of paracrine secretion. They can tolerate harsh environment and play an important role in treatment of intervertebral disc (IVD) [19]. Therefore, MSC EVs have a higher potential for treatment of IVDD compared with MSCs. Immunomodulatory effects and potential applications of MSC EVs and its advantages over MSC transplants in IVDD are presented in the current review.

## 2. EVs

EVs are structures released by eukaryotic cells and prokaryotic cells. EVs are vesicles enclosed by a lipid bilayer that cannot replicate and do not contain a functional nucleus.

**Figure 1:**
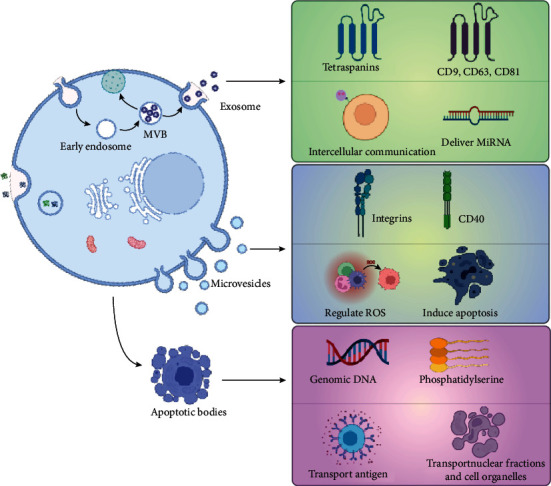
The origin, characteristic, and function of three main types of extracellular vesicles, including exosomes, microvesicles, and apoptotic bodies.

Studies have characterized three main types of extracellular vesicles including: exosomes, microvesicles and apoptotic bodies (Figure 1). Exosomes are small vesicles with a diameter of 50-200 nm, containing biomolecules such as microRNA (miRNA), which are most widely used and have significant medical application. Secretion of exosome is implicated in endocytosis and exocytosis. Early endosomes are formed by plasma membrane invagination, and then late endosomes are formed by vector selection. At last, multivesicular bodies (MVB) formed and fused with plasma membrane to release the substances contained in it referred as exosomes to the extracellular space [20–22]. Microvesicles are derived from plasma membrane and outward budding and a diameter ranging from 200 to 2000 nm, and they express integrins and CD40 [23, 24] and can regulate ROS and induce cell apoptosis. Apoptotic bodies originate from plasma membrane invagination and have a diameter ranging from 500 to 2000 nm and transport antigen, nuclear fractions, and cell organelles [23, 25, 26]. Although EVs are small in size, their surfaces are rich in lipids and proteins, including transmembrane/GPI-anchored extracellular protein and cytosolic/periplasmic protein with lipid or membrane protein-binding ability. Moreover, EVs carry functional molecules including deoxyribonucleic acid, ribonucleic acid, lipids, metabolites, cytoplasmic substances, and soluble extracellular proteins that are functionally active (such as cytokines, growth factors, and extracellular stroma) [21].

Several studies have explored the important roles of EVs. Previous studies report that EVs play key roles in immune response [27–29]. In addition to carrying proteins such as growth factors and cytokines, EVs can induce different signaling pathways, such as the Wnt and Notch pathways, through their surface ligands [30, 31], which are related to immune inflammation. Further, EVs can transfer and present antigen peptides. Therefore, EVs have high potential in vaccine preparation and use as diagnostic markers owing to their immunogenicity.

**Figure 2:**
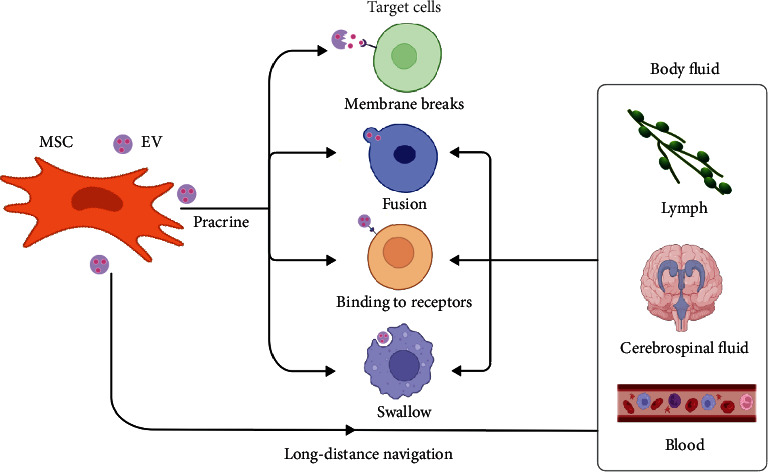
MSC EVs can carry out material transport and transmit signal to adjacent target cells by means of paracrine secretion. Alternatively, they can enter the blood, cerebrospinal fluid, lymph, and other body fluids for long-distance intercellular communication with target cells in other parts of the body.

EVs are involved in both long and short distance intercellular communication. After being secreted from the cell, a small portion of the EVs membrane ruptures to release its contents, such as growth factors, which are then used in adjacent cells [32]. In addition, EVs can travel between cells, moving to areas adjacent to tissues to function in paracrine way and also to distant body fluids, such as serum, lymph and cerebrospinal fluid, to intercellular communicate [33]. EVs can deliver cargo into the cytoplasm of target cells to perform intercellular communication through membrane fusion [34]. EVs can induce signaling by binding to many differentially exposed receptors on the surface of target cells [35–38]. Alternatively, EVs are transferred to the cytoplasm by endocytosis [39–41] and then discharge its cargo when it fuses with the endocytic membrane [36, 38]. By means of the above, EVs can transmit signal and transport substance to function as intercellular communicator (Figure 2).

Ability of EVs to encapsulate contents and transport them between cells has been widely explored [42–45]. Potential of EVs as drug carriers, known as “natural delivery systems, “ has received considerable attention. EVs are structures produced by the body naturally; thus, they are not easily attacked by the immune system and can circulate in the body for a long time. In addition, they can effectively cross natural barriers to transport drugs to the target site.

## 3. Characteristics and Functions of MSCs

MSCs are pluripotent nonhematopoietic stem cells derived from bone marrow (BM), umbilical cord (UC), placenta, amniotic fluid, fat, dental pulp, and induced pluripotent stem cells (IPSCs) or human embryonic stem cells (ESCs) [46, 47]. Primary BMSCs are plastic-adherent stromal cells with self-renewing ability, which have immunophenotypic strongly positive surface markers such as CD105, CD73, and CD90, but lack markers such as CD45, CD34, CD14, CD11b, and CD19. MSCs are characterized by multidirectional differentiation ability [48, 49], including chondrogenic ability which can be applied in intervertebral disc therapy.

MSCs have high regenerative ability and high immunomodulatory properties. Moreover, they can repair tissue damage and regulate cellular immunity by secreting bioactive substances including exosomes [50, 51]. Previous studies reported that MSCs promote development of immune cells mainly through cellular interactions. However, recent studies report that BMSCs also mediate their therapeutic function through the paracrine system [52, 53]. MSC transplantation has a positive effect on IVD regeneration. Hypoxia promotes differentiation of BMSCs into nucleus pulposus- (NP-) like cells. Notably, the structure of IVD has a unique microenvironment that is highly harsh compared with that of any other tissue. Acidic and high osmotic pressure environment of IVD can affect the function of transplanted cells and promote death of cells [2, 13, 54, 55].

Improved immune compatibility of MSCs has high potential in the field of disc therapy owing to the reduced risk of rejection. However, immunogenicity of BMSCs can be increased by exposure to inflammation and oxidative stress. This reduces their viability and differentiation ability, ultimately affecting the therapeutic application of BMSCs in the nucleus pulposus tissues which are already inflamed [56]. Nucleus pulposus is surrounded by a solid AF envelope. Therefore, it is difficult to transport a large number of MSCs into the NP without causing severe injury to the IVD, which significantly limits its therapeutic effect [15]. Therefore, MSC EVs have high potential as effective delivery vehicles in the intervertebral disc owing to their small size, low immunogenicity, and easy availability.

## 4. General Characteristics and Functions of MSC EVs

MSC EVs are EVs produced by the paracrine pathway of MSCs. MSCs secrete higher amounts of EVs compared with other types of cells [57]. Several experimental and clinical studies indicate that the therapeutic effect of bone marrow MSCs (BMSCs) is mainly mediated through their paracrine role, especially through EVs [17, 18, 58, 59]. MSCs EVs play immunomodulatory roles through paracrine effects thus transporting small biological molecules. MSC EVs carry complex cargoes similar to EVs, including proteins, nucleic acids and lipids [57]. However, MSC EVs can express tetraspanins such as common exosomal surface markers (CD81, CD63, and CD9) and adhesion molecules (such as CD29, CD44, and CD73) [57, 60]. As a result, MSC EVs play an indispensable role in cell communication, signal transduction, and cytokine delivery. Several growth factors and cytokines expressed by MSCs are transported by EVs as signal peptides; thus, they can be recognized by target cells. This makes MSC EVs an essential part of maintaining vitality and stability of their microenvironment [61].

MSC exosomes are cell-derived but have a very different composition from cell plasma. MSC exosomes are enriched in CD9, CD63, CD81, and HSP70, whereas GM130, calnexin, and cytochrome-C, which are expressed in MSC cells, are not expressed or are under-expressed in exosomes [62]. Zhang et al. explored miRNA profiles of adipose-derived MSC (ADSC) EVs and reported that 148 known miRNAs were present in ADSC EVs [63]. In addition, proteomic analysis of ADSC EVs showed presence of 1466 proteins associated with various cell functions [64]. High expression levels of extracellular proteins in MSC EVs relative to the cytoplasm protect cells affected by inflammation. Studies in leukemia using next-generation sequencing technology indicate that different types of miRNAs are significantly upregulated and downregulated in AML-derived BMSC EVs, implying that MSCs actively package miRNAs into EVs thus playing an immune-related role in disease [65]. These findings indicate that MSC EVs is not passively involved in delivery of cargos, but rather actively transport proteins and RNA from the cytoplasm to exert effects, including anti-inflammatory.

And MSC EVs have a double membrane structure that effectively insulates the contents from the harsh external microenvironment. Therefore, MSC EVs are promising drug-delivery systems for treatment of IVDD.

## 5. Therapeutic Application of Stem Cell-Derived EVs in Different Tissues

Inflammation, degradation of extracellular matrix, inhibition of cell proliferation, and oxidative stress are key characteristics of IVDD [5, 6, 66]. Therefore, inhibition of overexpression of inflammation, degradation of extracellular matrix, oxidative stress, and cell apoptosis plays a significant role in the treatment of IVDD.

**Figure 3:**
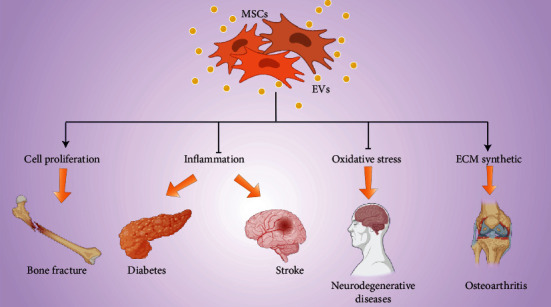
MSC EVs play a therapeutic role in a variety of diseases. MSC EVs repair fractures by promoting cellular cell proliferation, treat diabetes and stroke through anti-inflammatory therapy, treat degenerative neuropathy through antioxidative stress, and treat osteoarthritis by promoting extracellular matrix synthesis.

Several diseases exhibit similar immune characteristics to IVDD (Figure 3). High levels of inflammatory cytokines such as IL-1 *β*, TNF-*α*, and other inflammation-related factors affect insulin secretion and biosynthesis in diabetic patients [67]. Cord blood-derived MSC EVs and BMSCs EVs inhibit development of helper T1 (Th1) and Th17 cells and shift cytokine production from proinflammatory to anti-inflammatory cytokines. This shift restores the balance between Th1 and Th2 immune responses, thus improving therapy efficacy for type 1 diabetes [68, 69]. Moreover, EVs derived from human BMSCs alleviate type 2 diabetes by inhibiting inflammation [67].

MSC EVs can encapsulate mRNA and miRNAs, enabling MSCs to exert anti-inflammatory and immunomodulatory effects in a variety of tissues and organs [70]. EVs can change their miRNA profile according to the environment and participate in the recovery process of stroke by regulating inflammation by targeting the TGF-*β* signaling pathway [71–73]. MSC EVs carrying miRNAs can mediate immunity to slow disease progression by mediating oxidative stress in neurodegenerative diseases such as Alzheimer's disease and Parkinson's disease [74]. Liu et al. reported that human UCMSC EVs promote cell proliferation and migration by transferring miR-126 to promote fracture healing under hypoxic conditions [75]. Jing et al. reported that human UCMSCs incubated with kartogenin secrete miR-381-abanent EVs to target TAOK1 thus promoting chondrogenesis by upregulating expression of SOX9, aggrecan, and collagen II genes [76].

In summary, MSC EV agents have high potential in a wide range of diseases, and the therapeutic modalities for each disease may be different and specific, including inhibiting inflammation, inhibition of extracellular matrix decomposition, inhibition of oxidative stress, and promotion of cell proliferation. These phenotypic changes play a positive role in IVDD therapy.

## 6. Potential Applications of MSC EVs in IVDD

MSC EVs have high potential in treatment of IVDD. Bone marrow, cord blood, and adipose tissue are common sources of MSCs. MSCs from different sources have the ability to regenerate and secrete large amounts of EVs [75–85]. The microenvironment in degenerated IVD has low irrigation and high mechanical stress, low oxygen, high acid, and other factors [15]. Acidic environment can increase intake of EVs and further enhance the role of EVs in repairing original cells in the disc and promoting production of the extracellular matrix. These changes exert significant effect on maintaining the height of the disc and promoting restoration of the internal environment of the disc.

In addition, MSC EVs are natural components of the human body and have low immunogenicity. The lipid bilayer of MSC EVs enables them to overcome the harsh intervertebral disc microenvironment. The small size of MSC EVs allows them to enter the intervertebral disc in large quantities without eliciting an immune response. MSC EVs transport mRNAs, microRNAs, and proteins to regulate the changes in the IVD thus playing a role in maintaining microenvironment stability [86]. These features make EVs a good substitute to alleviate the limitations in IVDD treatment.

**Figure 4:**
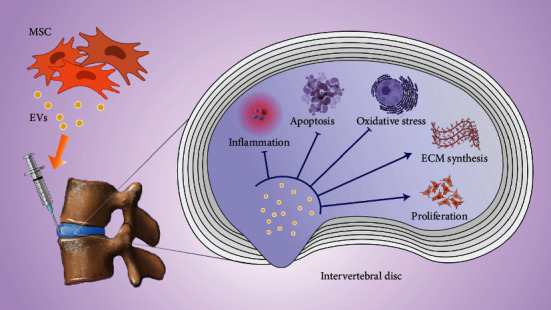
MSC EVs can play a role in anti-inflammatory, antiapoptotic, and antioxidative stress, extracellular matrix synthesis, and cell proliferation in degenerated IVD.

The main causes of IVDD include cell death, oxidative stress, loss of extracellular matrix, and accumulation of inflammatory cytokines. The mechanism of EVs in treatment of IVDD is described in the subsequent sections based on these aspects (Figure 4).

### 6.1. Antioxidative Stress

Overproduction of reactive oxygen species (ROS) is common in degenerative IVD. Oxidative stress promotes progression if IVD by regulating matrix metabolism, proinflammatory phenotype, apoptosis, autophagy, and disc cell senescence. Moreover, oxidative stress enhances matrix degradation and inflammation and reduces the number of viable and functional cells in the IVD microenvironment. In addition, ROS modifies matrix proteins in IVD, leading to oxidative damage of the extracellular matrix of the intervertebral disc and impairing of the mechanical structure and function of IVD [87]. Therefore, inhibition of oxidative stress is a potential mechanism of protecting the degenerated disc.

MSC EVs alleviate IVDD through exerting antioxidant and anti-inflammatory activities [88]. miRNA-31 found in MSC EVs play a significant role in inhibiting calcification in endplate chondrocytes (EPCs) under oxidative stress by targeting the ATF6-related ER-stress pathway. MSC EVs target ATF6-related oxidative stress pathway by delivering miR-31-5p in EPCs to inhibit apoptosis and calcification in EPCs under oxidative stress thus improving the symptoms of IVDD rat model [89]. And Hu has reported that BMSC EVs could reduce ROS and alleviate the inhibitory effect of compression on NP cells proliferation and viability by inhibiting oxidative stress [90].

### 6.2. Anti-Inflammatory

NP and annulus fibrosus cells, as well as immune system cells (such as macrophages, monocytes, dendritic cells, B lymphocytes, and NK cells), comprise several proinflammatory molecules, leading to changes in the microenvironment of IVD, thus indirectly causing degeneration. Cytokines trigger a series of pathogenic responses that lead to autophagy, senescence, and apoptosis in the IVD [91–94]. NF-*κ*B pathway, MAPK pathway, and Notch pathway are closely related to expression levels of interleukin-1 (IL-1) and tumor necrosis factor-*α* (TNF-*α*), which are key factors implicated in IVDD [6]. Therefore, suppression of inflammation, regulation of immunity, and restoration of homeostasis is essential for treatment of IVDD.

Shim et al. reported that EVs secreted by MSCs downregulated expression of various proinflammatory cytokine genes which are associated with degeneration of NP in a coculture of MSCs from the vertebral body and NPCs [14]. Chen reported that NP cells take up BMSC EVs and suppress expression of H_2_O_2_-induced inflammatory markers such as iNOS and IL-6. Moreover, BMSC EVs suppress H_2_O_2_-induced NLRP3 inflammasome activation in NP cells and dampen activation of inflammatory factors such as caspase-1, IL-1*β*, TXNIP, and NLRP3 [88]. Human embryonic MSC EVs increase M2 macrophage infiltration and decrease infiltration of M1 macrophages and M1-associated cytokines, IL-1*β*, and TNF-*α* in cartilage tissues [95]. The effect of human embryonic MSC EVs on IVD may be similar to the effect on cartilage tissue owing to the similarity between IVD and cartilage tissue.

### 6.3. Extracellular Matrix Degradation and Synthesis

Extracellular matrix degradation plays an important role in etiology of IVDD. Inhibition of matrix degradation is an important approach for preventing matrix loss during cell degeneration and can be used to treat the diseased system to protect tissue engineering structures. Therefore, inhibition of matrix degradation and promotion of its synthesis has practical therapeutic implications.

MSCs can effectively promote expression of collagen II and chondroitin sulfate (CS) which are implicated in NP extracellular matrix synthesis [96]. Notably, treatment of NPCs with BMSC EVs upregulated expression of anabolic/matrix protective genes in NPCs such as aggrecan, collagen II, and sox-9. On the contrary, BMSC EVs downregulated expression of matrix-degrading genes (MMP-1 and MMP-3) [97]. These findings indicate that BMSC EVs maintain stability of the matrix in NP cells and protect it from dysregulated gene expression. IVD is characterized by several progenitor cells. BMSC EVs can recruit progenitor cells to the degenerated IVD and induce them into NP cells thus replacing the lost cells and promoting formation of extracellular matrix [97]. Human embryonic MSC EVs highly express CD73/Exto-5′-nucleotidase, which converts extracellular AMP to adenosine. Adenosine induces prosurvival AKT and ERK signaling through interaction with adenosine receptors to further promote cell proliferation, migration, and extracellular matrix secretion [95]. These findings indicate that MSC EVs can be used for treatment of IVDD by increasing the relative amount of extracellular matrix.

### 6.4. Antiapoptotic and Regenerative Activities of MSC EVs

Abnormal apoptosis-induced np cell death can lead to disorders in metabolism of extracellular matrix in the nucleus pulposus of the intervertebral disc. Therefore, interventions that target apoptosis of NP can inhibit progression of IVDD [98]. Moreover, supportive cell proliferation increases the relative volume of nucleus pulposus cells as a therapeutic strategy for IVDD [99]. Therefore, studies on NP cell apoptosis and regeneration can improve understanding of IVDD and provide a basis for development of potential therapeutic strategies.

Recent studies report that MSC EVs play an important role in inhibiting apoptosis of NP cells. The effect of BMSC EVs on H_2_O_2_-induced apoptosis of NP cells was previously explored. The levels of apoptotic proteins such as caspase-3 and caspase-9 increased after H_2_O_2_ treatment but the expression of these proteins was downregulated by BMSC EV treatment [88]. BMSC EVs increase the level of autophagy in NPCs and decrease apoptosis level by upregulating the miR-155 expression, which targets Bach1 in NPCs and promotes expression level of HO-1 thus abrogating IVDD [100].

Studies report that MSC EVs play a role in regeneration in IVDD. Previous findings indicated that PCNA+ cells were significantly increased, and CCP3+ cells were significantly decreased in cartilage tissue in the exosome-treated group after 12 weeks compared with the control group. This indicated that human embryonic stem cell-derived MSC EVs promoted proliferation of chondrocytes. MSC EVs can promote proliferation of NP cells and reduce apoptosis in IVDD owing to the similarity between IVD and cartilage tissue [95]. Notably, previous findings from CCK8 assay showed that the NPC proliferation rate increases with increase in the duration of interaction after treatment of BMSC EVs [97].

## 7. miRNA of MSC EVs Treating IVDD

MicroRNAs (miRNAs) is a class of short, endogenously initiated noncoding RNA. They are involved in regulation of gene expression at the posttranscriptional level through recognition of cognate sequences and interference of transcriptional, translational, or epigenetic processes [101, 102]. As components of EVs that play a major role in intercellular communication, miRNAs affect cell survival by altering the levels of components essential to life [103]. Katsuda and Ochiya reported that MSC exosomal miRNAs are associated with several MSC EV-mediated cellular activities, such as antiangiogenesis, antiapoptosis, immunomodulation, and antifibrosis [104]. This indicates that MSC EVs can be used as vehicles for gene transmission, thus exerting a positive effect on IVDD by delivering miRNA.

### 7.1. MiR-23

A previous study showed that high expression level of miR-23a-3p in human BMSCs EVs can promote cartilage regeneration and heal cartilage defects in vivo by increasing PTEN levels and upregulating AKT expression [105]. Moreover, Mir-23a-3p carried passively by MSC EVs can promote migration, proliferation, and differentiation of chondrocytes.

### 7.2. MiR-140

EVs derived from miR-140-5p-overexpressing synovial mesenchymal stem cells induce chondrocyte proliferation and migration without reducing ECM secretion. This implies that EVs can exert a similar effect on NPCs as that observed in articular cartilage. Furthermore, miR-140-5p promotes matrix synthesis by upregulating SOX9 and aggrecan expression through targeted inhibition of RALA [106]. Notably, miR-140 (miR-140-3p) inhibits apoptosis by regulating the regenerative role of KLF5/N-cadherin/MDM2/Slug axis in IVDD [107]. Studies report that miR-140 exerts beneficial effect on chondrocytes and cartilage matrix. MSC EVs are promising cargo carriers for miR-140 delivery for treatment of osteoarthritis by remodeling cartilage matrix without any unwanted immune responses [108].

### 7.3. MiR-25

Recent studies indicate that miR-25-3p-overexpressing BMSCs secrete miR-25-3p through EVs [109]. Notably, miRNA-25-3p delays progression of IVDD by inhibiting IL-1*β*-induced inflammation effects [110]. In addition, miR-25-3p regulates cartilage homeostasis by targeting ECM degradation and is thus a potential therapy for IVDD [111]. MiR-25 protects NPCs against apoptosis in IVDD by targeting SUMO2 [112]. These findings indicate that EVs derived from mir-25-3p-overexpressing BMSCs have high potential for development of IVDD therapy.

### 7.4. MiR-142

BMSC EVs actively packaged with miR-142-3p inhibit MAPK signaling by targeting MLK3 thus alleviating IL-1-mediated inflammatory injury of NPCs [113]. Furthermore, miR-142-3p promotes expression of HMGB1, induces proliferation and migration of cartilage endplate (CEP) cells, and inhibits apoptosis of CEP cells by promoting autophagy [114]. Moreover, miR-142-3p inhibits activation of NF-*κ*B and JNK pathways thus inhibiting inflammation and apoptosis of NPCs in degenerated IVD [113, 115].

## 8. Discussion

### 8.1. Prospects

Several effective drugs for degenerative diseases have been developed in the recent past which makes treatment of degenerative diseases more diversified. However, most advanced drugs do not reach their targets owing to the harsh microenvironment. Thus, efficient drug vehicles should be developed to transport the drug through the microenvironment while maintaining its stability and efficient delivery to its target.

EVs can be used to circumvent the limitations of traditional drugs, including poor water solubility, poor biocompatibility, low permeability to cells, unsatisfactory distribution, and effective elimination in vivo. EVs have a natural targeting ability and high biological barrier permeability; thus, they can efficiently deliver drugs to their targets.

Biocompatibility of MSC-EV plays a significant role in minimizing adverse manifestations including immune reactions [116–118]. They deliver cargo to target cells through a series of surface adhesion proteins and carrier ligands. Moreover, MSC EVs can effectively permeate cell membranes to deliver their contents to target cells in vivo. In addition, EVs can deliver a variety of bioactive substances and easy-to-deactivate or easily degradable ingredients through multiple pathways and sites. They safely transfer these substances to target cells thus modulating several processes such as tissue repair and immune response [119]. Nanotechnology has high potential as it is characterized by targeting of drugs to specific sites and controlled-release of drugs to minimize side effects. EVs combine the advantages of nanomaterials and biological delivery system thus improving drug delivery [120]. EVs show great therapeutic potential when used in combination with hydrogel, thus timely transport of cargos with short half-lives can be achieved in vivo. Long-term retention and controlled release of extracellular vesicles in vivo is a novel research approach for treatment of IVDD using combinations of EVs and hydrogels.

MSCs are characterized by several limitations when directly transplanted in vivo. Nongenetically modified bone marrow mesenchymal stem cells may exhibit chromosomal abnormalities during an early passage, resulting in formation of malignant tumors [121]. Notably, studies have not explored the tumorigenesis potential of MSC-EVs [122]. Acquisition and amplification of MSCs result in mixing with other cells, resulting in low purity and cellular senescence during amplification process. Transplanted MSCs decrease with decrease in metabolic level, and their repairability is weakened over time [123]. In vitro ability of MSCs to differentiate and proliferate decreases gradually in culture [124, 125]. These limitations affect survival and differentiation of MSCs. Moreover, the actual number of NPCs differentiated from MSC in the IVD after MSC transplantation is not high. Recent studies report that MSC transplantation may not secrete products that inhibit inflammatory responses during MSC transplantation in IVD [126].

MSC EV therapy is an effective alternative for traditional MSC therapy for repair of IVDD. Paracrine activity of MSCs plays a major role in therapeutic effect compared with use of MSCs s [127]. EVs are significantly relative to MSCs in volume and are suitable for use in narrow IVD environments, implying that transplanting EVs into IVD potentially causes less damage compared with implanting MSCs [128]. In addition, MSCs can produce continuously abundant EVs when making immortal cell lines. This implies that MSCs can consistently and repeatedly produce EVs for experimental and clinical applications. Furthermore, MSCs can be modified to upregulate expression of specific miRNAs in the EVs, this abrogating progression of IVDD [129]. Moreover, EVs have potential ability for tissue regeneration. Studies report that MSC EVs present high potential for treatment of osteoarthritis [130]. Notably, MSC EVs enhance periodontal ligament cell function and stimulate periodontal regeneration [131]. MSC EVs promote proliferation, migration, and tenogenic differentiation of tendon stem or progenitor cells [132]. Therefore, further studies should explore the regenerative capacity of MSC-EV on intervertebral disc tissue.

### 8.2. Limitations of Application of MSC EVs

MSC EVs have been used clinically in some fields. MSC EVs can be used as early prediction tools for preclinical trials. Further, they are used to effectively detect drug effects in humans [133]. However, application of MSC EVs is associated with a few limitations; thus, they are not widely used in clinical trials.

Firstly, a unified standard should be formulated when extracting and storing EVs. A major challenge in converting MSC-EV formulations into experimental therapeutic products is determining the specific ability or capability of a product to achieve a particular biological effect which is referred as the potency metrics [134]. Development of quantifiable, robust, and reproducible parameters to predict the therapeutic efficacy of MSC-EVS can help address this issue in analysis of MSC-EVs [135]. Studies have found that the biological activity and the morphology of EVs will still be impacted in 4 weeks when stored in a comprehensive storage way. Standardized procedures for isolating and storing EVs still need to be developed customized for EV matrix and application technologies, reagents and storage containers, and storage times [136]. Therefore, developing the storage stability of EVs from multiple perspectives, especially the long-term stability, is significant.

Secondly, the reported efficacy of MSC-EVs formulations for multiple diseases is complex and varied. Thus, to develop potency analyses, it is important to identify and quantify the most relevant EV properties expected to be biologically active in EV-mediated therapy [137]. Some studies have found that EVs rely on their miRNA for therapeutic effects, and since EVs carry many different types of miRNAs, the role of miRNAs of various EV sources in the intervertebral disc should be explored further.

Thirdly, in vivo safety of MSC EVs should be determined in preclinical trials. Further, clinical use of MSC EVs should be explored further.

## 9. Conclusion

Cell therapy is widely used in treatment of IVDD. Previous studies reported that MSCs alleviated IVDD through differentiation of MSC cells into NPC cells, which promoted regeneration of IVD. However, recent studies report that paracrine activity of MSCs plays a major role in alleviating IVDD. MSC EVs are products of MSC secretion that can perfectly replace MSC in development of IVDD therapy. MSC EVs are promising products in acellular therapy. They can secrete several factors thus promoting cell proliferation and antiapoptosis, regulating the microenvironment, and thus reducing the impact of factors such as inflammatory cues in IVDD. MSC EVs can adapt to the harsh microenvironment of IVDs, overcoming in the limitations of MSC transplantation. Further, MSC EVs can be used in drug delivery to maintain the stability of the drug in the harsh microenvironment of IVD. In summary, MSC EVs play a significant role in treatment of IVDD.

## Data Availability

Data sharing is not applicable to this article as no new data was created or analyzed in this study.
